# Regadenoson is a better myocardial vasodilator than dipyridamole in normal volunteers, but the data is less compelling in patients

**DOI:** 10.1186/1532-429X-13-S1-P124

**Published:** 2011-02-02

**Authors:** Sujethra Vasu, W Patricia Bandettini, Li-Yueh Hsu, Peter Kellman, Marcus Y Chen, Joel Wilson, Steve Leung, Sujata M Shanbhag, O Julian Booker, Christine Mancini, Jennifer Henry, Tracy Lowrey, Andrew E Arai

**Affiliations:** 1 National Institutes of Health, Bethesda, MD, USA

## Introduction

Regadenoson is a selective Adenosine-2A receptor agonist and is used for myocardial perfusion imaging. Dipyridamole causes indirect vasodilation by inhibiting cellular reuptake of adenosine. The purpose of this study was to assess whether regadenoson is a better coronary vasodilator than dipyridamole in normal volunteers and in patients.

## Hypothesis

Regadenoson causes a 25% higher myocardial blood flow (MBF) than dipyridamole.

## Methods

Forty patients with normal coronaries/minimal stenosis on coronary CT angiography had also undergone vasodilator stress MR (regadenoson n=20, dipyridamole n=20). Seventeen healthy normal volunteers with Framingham score less than 1% underwent vasodilator stress testing with regadenoson and dipyridamole in two separate studies using a SSFP sequence. Stress imaging was done 70 seconds post regadenoson injection and 4 minutes after completing the dipyridamole infusion. All patients and volunteers received aminophylline after stress imaging. Rest imaging was done 20 minutes later. MBF in ml/min/g and Myocardial perfusion reserve (MPR) were quantified using a fully quantitative model constrained deconvolution (MCD).

## Results

**Figure 1:**
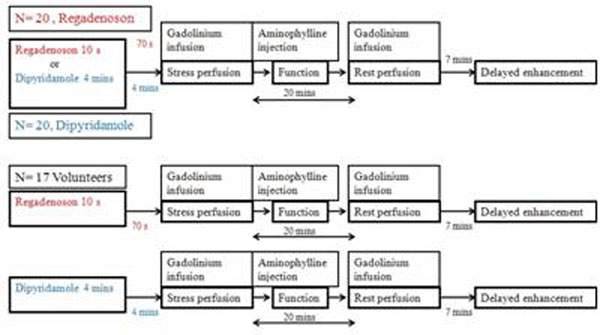
Study design

**Figure 2:**
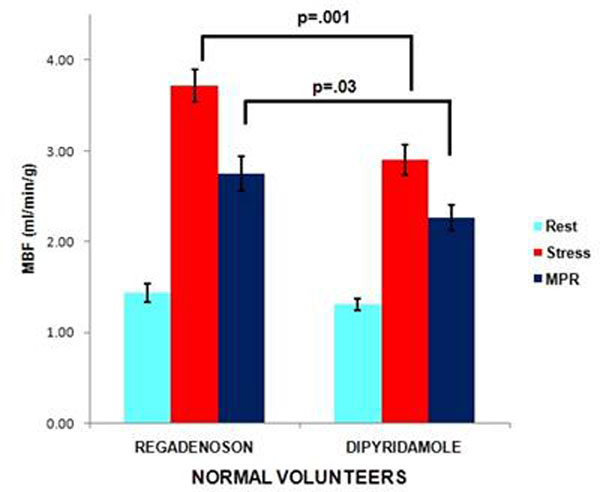
Rest, Stress MBF and MPR in Normal volunteers

**Figure 3:**
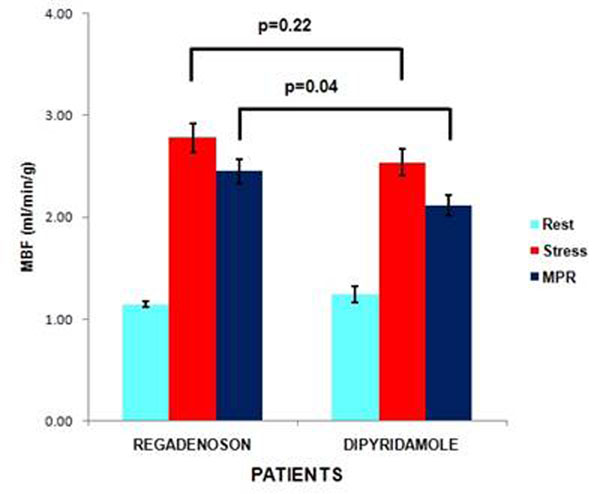
Rest, Stress MBF and MPR in Patients

Normal volunteers had higher stress MBF (mean ± standard error) with regadenoson than dipyridamole (3.72± 0.18 vs. 2.90± 0.17, p=0.001) and higher MPR with regadenoson (2.75± 0.19 vs. 2.27±0.14, p=.03). In patients, higher MPR was found with regadenoson (2.45 ± 0.12, vs. 2.12 ±.10, p=.04). Stress MBF trended higher in patients with regadenoson than dipyridamole (2.78± 0.14 vs. 2.54± 0.13, p=0.22). No difference in the resting blood flow between regadenoson and dipyridamole was found in normal volunteers (1.44± 0.19 vs. 1.31±0.07, p=.07) and in patients (1.14±0.03 vs. 1.24±0.08, p=.26) respectively. Figures 1-3

## Conclusions

Regadenoson is a better coronary vasodilator than Dipyridamole in normal volunteers with higher MBF and MPR. However in patients, the small sample size in this study limits the sensitivity to detect differences in stress perfusion.

